# Editorial: Cytokine dynamics in livestock: from health to pathology

**DOI:** 10.3389/fvets.2025.1732449

**Published:** 2025-11-14

**Authors:** Mohanned Naif Alhussien, Anja Serap Sipka, Giovanna De Matteis, Susana Flores-Villalva, Jamal Hussen

**Affiliations:** 1 Reproductive Biotechnology, School of Life Sciences, Technical University of Munich, Freising, Germany; 2 Department of Population Medicine and Diagnostic Sciences, College of Veterinary Medicine, Cornell University, Ithaca, NY, United States; 3 Research Centre for Animal Production and Aquaculture, Council for Agricultural Research and Economics, CREA, Monterotondo, Italy; 4 National Centre for Disciplinary Research in Animal Health and Safety, National Institute for Forestry, Agricultural and Livestock Research (INIFAP), Mexico City, Mexico; 5 Department of Microbiology, College of Veterinary Medicine, King Faisal University, Al-Ahsa, Saudi Arabia

**Keywords:** cytokines, immunoregulation, livestock health, disease resistance, nutritional immunity, reproductive immunology, diagnostic biomarkers

Cytokines are pivotal signaling molecules that coordinate the complex interplay between innate and adaptive immune systems in livestock. These molecules including interleukins, chemokines, interferons, lymphokines, tumor necrosis factors, growth factors, and adipokines are fundamental regulators of immune homeostasis, inflammation, and host defense against pathogens and environmental stressors (1, 2). Acting in autocrine, paracrine, or endocrine modes, cytokines influence diverse physiological processes, including immune cell activation and differentiation, tissue remodeling, reproductive function, lactation, and metabolic balance during physiological transitions (3, 4). Despite their central role in immunity and health, the regulatory networks and species-specific functions of cytokines in livestock remain incompletely characterized. Important questions persist regarding how these mediators shape immune resilience, disease susceptibility, and productivity in different species. To address these gaps, the Research Topic “*Cytokine Dynamics in Livestock: From Health to Pathology”* was launched to consolidate recent advances in cytokine-driven mechanisms of immune regulation, hos-pathogen interactions, and physiological adaptation in livestock.

This Research Topic comprises seven original research articles, covering a wide range of physiological and pathological contexts, from reproductive and nutritional regulation to host responses against bacterial and mycobacterial infections. Collectively, these studies advance our mechanistic understanding of cytokine biology and highlight the translational potential of cytokine-based diagnostics, therapeutics, and management strategies to enhance livestock health and productivity.

## 1 Cytokine regulation in nutritional and reproductive physiology

Two studies in this Research Topic provide mechanistic insights into how physiological and nutritional interventions modulate cytokine networks in cattle. Somagond et al. demonstrated that repeated parenteral supplementation with combined trace minerals and vitamins during the transition period significantly enhanced mammary innate immunity and improved colostrum composition in multiparous crossbred cows. This intervention shifted cytokine profiles toward an anti-inflammatory state by reducing the concentration of pro-inflammatory mediators (IL-1β, IL-6, IL-8, IL-17A, IFN-γ) and increasing anti-inflammatory cytokines (IL-4, IL-10), concomitant with reduced oxidative stress and improved udder health. These results emphasize the critical role of micronutrient balance in modulating cytokine-mediated immune resilience, particularly during the periparturient period when cows are highly vulnerable to infection and inflammation.

In the reproductive context, Yousef et al. showed that platelet-derived secretions modulate uterine immunity in cattle by acting on polymorphonuclear neutrophils (PMNs) and endometrial epithelial cells (BEECs). Platelet-conditioned media suppressed pro-inflammatory cytokines (TNFA, IL1B) while enhancing anti-inflammatory mediators such as prostaglandin E2 (PGE2), lipoxin A4 (LXA4), and TGFB1. These coordinated cytokine-lipid responses reprogrammed immune and epithelial cells toward a pro-resolving state, establishing an anti-inflammatory uterine environment supportive of implantation and early pregnancy. The study reveals a novel platelet-driven mechanism that shapes uterine cytokine networks and highlights the potential of platelet derivatives as modulators of reproductive immune balance in cattle.

## 2 Cytokine responses in infection and inflammation

Studies in this Research Topic highlight significant advances in understanding the immunopathological and diagnostic roles of cytokines during infectious diseases in livestock. Flores-Villalva et al. employed multicolor flow cytometry to analyze antigen-specific cytokine responses of CD4^+^ T lymphocytes in buffaloes and cattle naturally exposed to *Mycobacterium bovis*. Their results revealed broadly similar immune profiles across species but also distinct enrichment of IL-17A^+^, IFN-γ^+^IL-17A^+^, and TNF-α^+^IL-17A^+^ subsets in buffaloes. Notably, the frequencies of IFN-γ^+^ and TNF-α^+^ T cells closely correlated with infection status, underscoring the diagnostic potential of cytokine polyfunctionality and providing mechanistic insight into interspecies differences in immune adaptation to bovine tuberculosis. Franzoni, Signorelli, Mazzone et al. (2024) assessed cytokine signatures as diagnostic biomarkers of *M. bovis* infection in Mediterranean buffaloes. Infected animals exhibited elevated concentrations of IFN-γ, IL-17, IL-10, TNF-α, IL-1α, IL-6, and MIP-1β following stimulation with purified protein derivative of *Mycobacterium bovis*, while discriminant analysis identified IL-10, TNF-α, IL-1α, IL-6, and MIP-1β as the most informative markers. In a related investigation, Franzoni, Signorelli, Donniacuo G. et al. (2025) examined cytokine responses to *Brucella* antigens in buffaloes and demonstrated that infected animals showed increased IFN-γ, IP-10, and MCP-1 levels, whereas IL-1α, IL-1β, IL-6, and IL-10 predominated in healthy controls. A composite cytokine profile comprising IFN-γ, IP-10, IL-1α, IL-1β, and IL-6 effectively differentiated infected from non-infected animals, improving diagnostic precision beyond conventional serological assays.

Rouault et al. investigated plasma cytokine and chemokine responses during natural outbreaks of bovine respiratory disease (BRD) in feedlot bulls. Although no single cytokine profile could discriminate infected from healthy animals, elevated IL-17A and IFN-γ levels were associated with greater disease severity and relapse risk. The study also highlighted the influence of preconditioning strategies, such as vaccination and antibiotic prophylaxis, on cytokine responses, underscoring how management practices can modulate immune trajectories and disease outcomes during BRD outbreaks. Satheesan et al. conducted transcriptomic profiling of milk somatic cells in *Bos indicus* (Sahiwal) cows to examine cytokine and chemokine dysregulation during mastitis. The analysis revealed broad immune reprogramming, with upregulation of IL-17 signaling, Th1/Th2 differentiation, and phagosome pathways, alongside suppression of cytokine-cytokine receptor and chemokine signaling. Downregulation of key chemokines (CCL2, CCL8, CXCL10) suggested impaired immune cell recruitment and weakened mammary defense, while altered expression of IFN-related genes and TNF superfamily members indicated cytokine imbalance. These findings highlight dysregulated cytokine-chemokine networks as central to mastitis immunopathogenesis and potential targets for improving mammary immune resilience through immunogenetic or therapeutic interventions.

## 3 Broader perspectives and concluding remarks

The studies compiled in this Research Topic collectively highlight the multifaceted roles of cytokines as central regulators and biomarkers of immune competence in livestock. Across nutritional, reproductive, and infectious contexts, cytokine dynamics emerge as critical determinants of immune balance, resilience, and disease resistance. Advances in multiplex immunoassays, flow cytometry, and transcriptomic profiling have revealed species- and condition-specific cytokine signatures that can serve as powerful biomarkers for diagnosis, prognosis, and therapeutic monitoring. Looking ahead, integrating cytokine profiling with next-generation systems immunology approaches such as single-cell sequencing, genome editing, and multi-omics will deepen our understanding of the regulatory networks governing livestock immunity. Translating these insights into applied strategies offers opportunities to refine diagnostics, develop cytokine-targeted interventions, and strengthen immune-informed herd management. Collectively, this body of work underscores the pivotal importance of cytokines in safeguarding animal health and productivity while setting the stage for future innovations in immunomodulation and livestock welfare.
